# HER2 splice variants in breast cancer: investigating their impact on diagnosis and treatment outcomes

**DOI:** 10.18632/oncotarget.27789

**Published:** 2020-11-17

**Authors:** Vic Hart, Hannah Gautrey, John Kirby, Alison Tyson-Capper

**Affiliations:** ^1^Translational and Clinical Research Institute, Faculty of Medical Sciences, Newcastle University, Newcastle upon Tyne, UK

**Keywords:** HER2, Δ16-HER2, splice variant, breast cancer, trastuzumab

## Abstract

Overexpression of the HER2 receptor occurs in approximately 20% of breast cancer patients. HER2 positivity is associated with poor prognosis and aggressive tumour phenotypes, which led to rapid progress in HER2 targeted therapeutics and diagnostic testing. Whilst these advances have greatly increased patients’ chances of survival, resistance to HER2 targeted therapies, be that intrinsic or acquired, remains a problem.

Different forms of the HER2 protein exist within tumours in tandem and can display altered biological activities. Interest in HER2 variants in breast cancer increased when links between resistance to anti-HER2 therapies and a particular variant, Δ16-HER2, were identified. Moreover, the P100 variant potentially reduces the efficacy of the anti-HER2 therapy trastuzumab. Another variant, Herstatin, exhibits ‘auto-inhibitory’ behaviour. More recently, new HER2 variants have been identified and are currently being assessed for their pro- and anti-cancer properties.

It is important when directing the care of patients to consider HER2 variants collectively. This review considers HER2 variants in the context of the tumour environment where multiple variants are co-expressed at altered ratios. This study also provides an up to date account of the landscape of HER2 variants and links this to patterns of resistance against HER2 therapies and treatment plans.

## INTRODUCTION

### Human epidermal growth factor receptor

Breast cancer occurs in 1 in 8 women within their lifetime and is the second highest cause of cancer related deaths in the UK [1, 2]. Breast cancer is an umbrella term for a highly heterogeneous group of breast tumours. Human Epidermal Growth Factor Receptor 2 positive (HER2+) breast cancer is a subgroup defined by over-expression of the HER2 tyrosine kinase receptor, which transduces biochemical signals across the cell membrane and amplification of the HER2 gene, *ErBb2* [3]. *ErBb2* is located on chromosome 17q21 and was identified in the 1980s due to its overexpression in a human mammary carcinoma [4]. HER2 positivity occurs in 20–30% of breast cancers [5].

**Figure 1:**
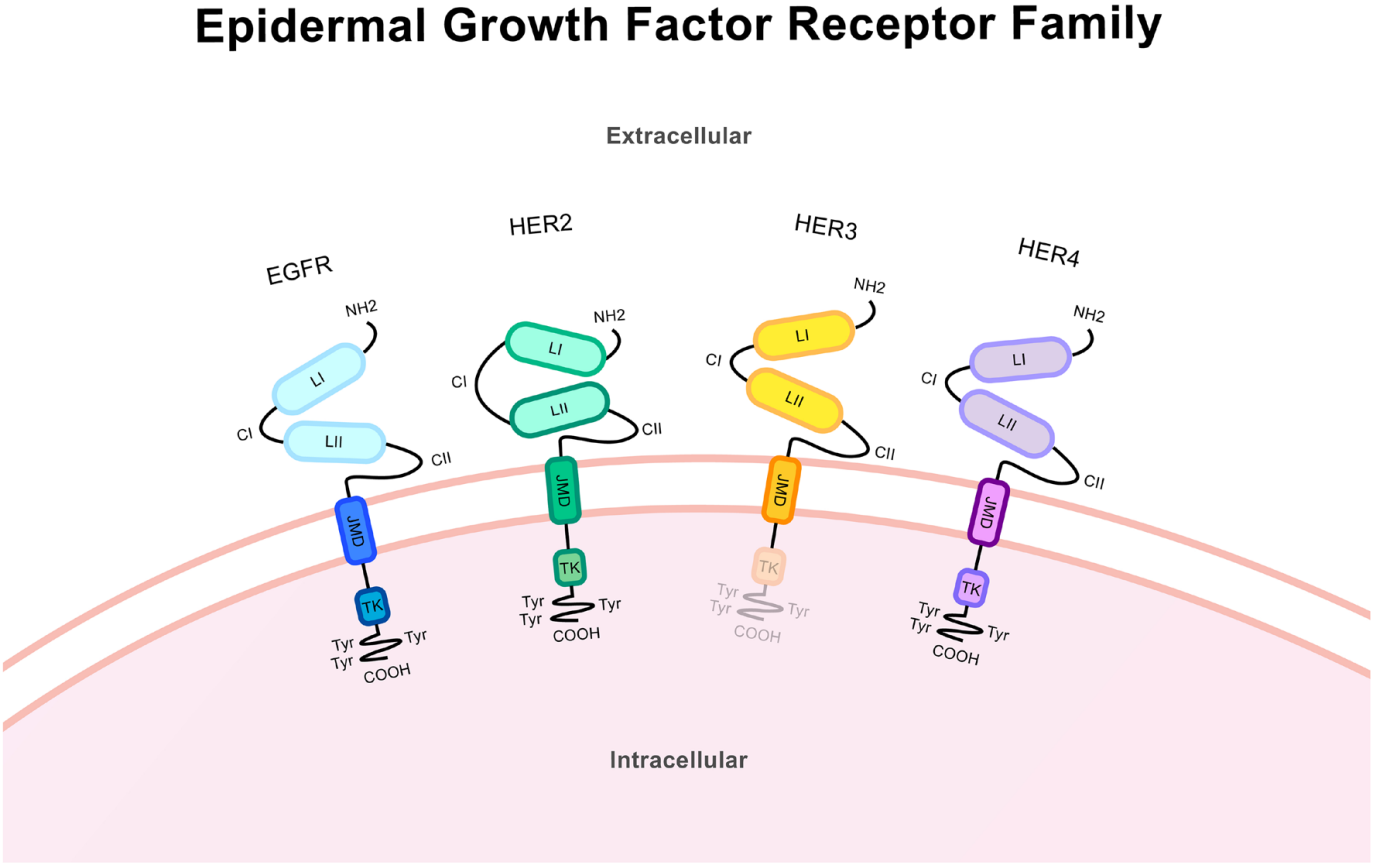
Protein structure of the epidermal growth factor receptor family. These structures from the N-terminus (NH2) to the C-terminus (COOH) includes a large extracellular ligand-binding region, a single transmembrane domain spanning the cell membrane and an intracellular domain. The extracellular region includes the ligand-binding domains LI and LII and the cysteine-rich domains CI and CII. Across the cell membrane lies the juxtamembrane domain (JMD). The intracellular region includes the tyrosine kinase domain (TK) and trans-phosphorylation sites/tyrosine residues and tyrosyl phosphorylation sites (Try) in the C-terminal region. Receptor specificity comes from variations in the kinase and the C-terminal domains. EGFR and HER4 contain the complete structure of the kinase receptor. HER3 lacks a functional tyrosine kinase domain and HER2 is an orphan receptor and as such has no known ligands. HER2 has an altered extracellular conformation and constitutively activated shape.

HER2 is a member of the epidermal growth factor receptor family which includes EGFR, HER2, HER3 and HER4 (Figure 1). HER2 consists of a larger extracellular region, a transmembrane domain, and an intracellular region including the tyrosine kinase domain [6]. Receptor activation by HER proteins occurs upon extracellular ligand binding, excluding HER2 which has no known ligands and instead maintains a constitutively activated shape and is thus called an ‘orphan receptor’. Ligands include many growth factors and heregulins (also called neu differentiation factors) [7]. Hetero- or homo-dimerization then occurs and shifting of the receptor domains allows for the auto-phosphorylation of C-terminal tyrosine residues. As the dimerization is asymmetrical the kinase domain from one receptor allosterically activates the other *via* this phosphorylation [8]. These C-terminal tyrosine residues act as docking sites, providing the location for the subsequent activation of downstream signalling molecules. Hetero-dimerization occurs between any of the EGFR family members with HER2 being the favoured dimer partner [6, 9]. Shedding of the extracellular domain (ECD) can occur through cleavage performed by matrix metalloproteases, leaving only the intracellular domain (ICD) [10, 11]. This can mediate signalling by altering dimer formation [12, 13].

**Figure 2:**
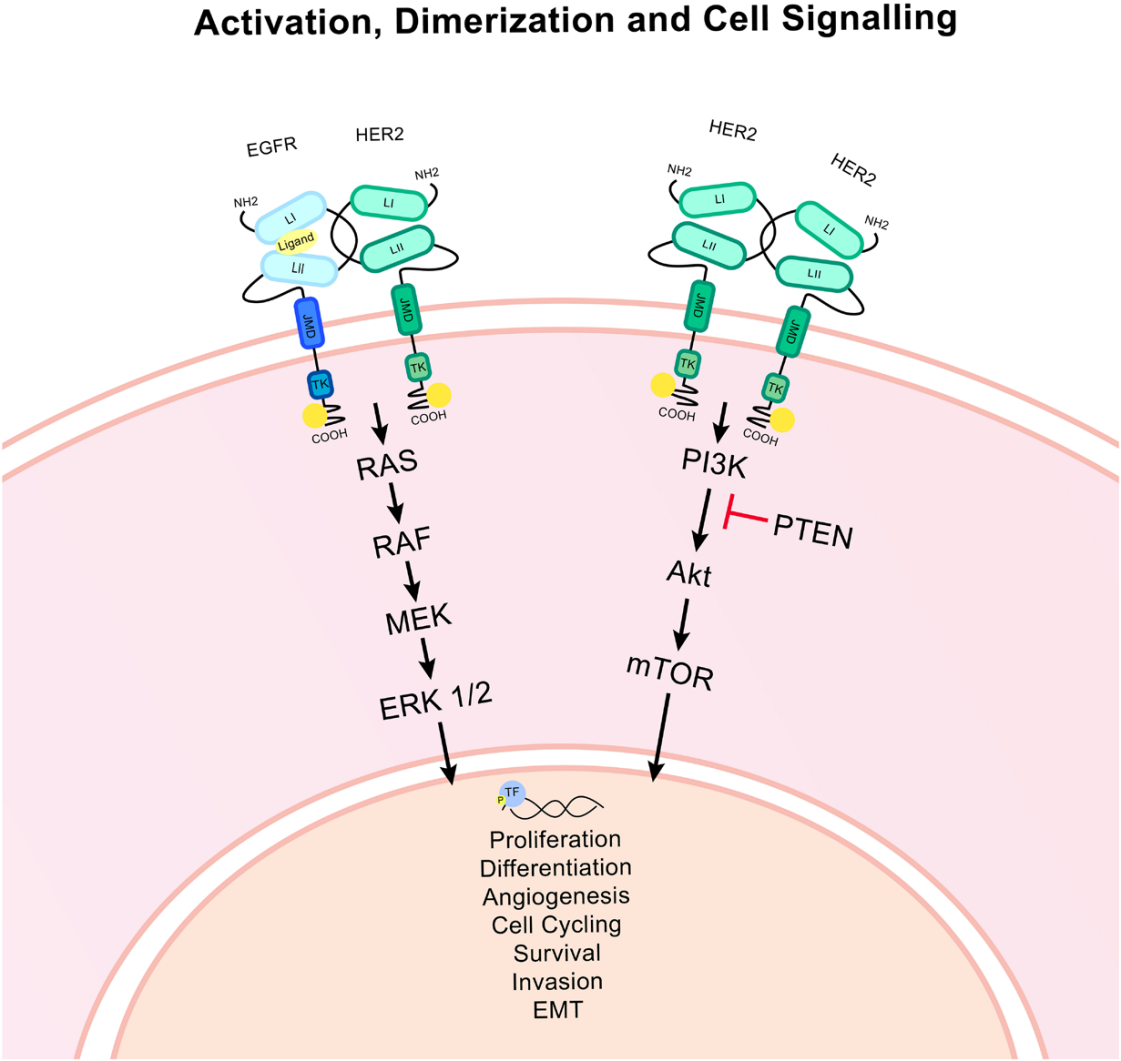
HER2 associated signalling pathways. Ligand binding to an EGFR/HER2 heterodimer causes the activation of signalling pathways *via* the phosphorylation of the tyrosine kinase domain of the intracellular HER2 molecule. The HER2 homodimer is constitutively activated. The RAS/MAPK and PI3K/Akt pathways are activated which culminates in the activation of important downstream effectors and transcription factors (TF). Ultimately this signalling can alter transcription and modulate gene expression, impacting upon many important cellular functions. Epithelial to mesenchymal transition is denoted as EMT. PTEN is an important inhibitor of the PI3K/Akt pathway. Arrows indicate signalling.

HER2 drives tumour progression *via* cellular signalling pathways (Figure 2). The rat sarcoma/mitogen-activated protein kinase (RAS/MAPK) pathway culminates in altered expression of genes related to proliferation, apoptosis and differentiation translating amplified signalling through HER2 into increased cancer progression [14]. ERK 1/2 (p44/p42) is a key node in this pathway and is activated *via* phosphorylation. This pathway also is linked to the regulation of the E2F activator transcription factors which control entry into the S phase of the cell cycle [15, 16].

The phosphatidylinositol 3-kinase (PI3K)/Akt pathway also triggers the phosphorylation of transcription factors that impact upon the regulation of apoptosis, metabolism, protein synthesis, angiogenesis, epithelial-mesenchymal transition (EMT) and proliferation [17]. Many other pathways are known to be affected by HER2 signalling including notch, Wnt, Src family kinases and various transcription factor families [18].

HER2 signalling is greatly impacted upon by its dimerization pattern with other EGF family members [19]. EGFR phosphorylation and activation is driven by HER2/EGFR dimerization as well as homo-dimerization. The HER2/HER3 dimer is responsible for most of the activation of the HER3 receptor as HER3 has an inactive kinase domain. This dimer is important for PI3K/Akt signalling, particularly in HER2+ tumours [20]. These dimerization pairings contribute greatly to HER2 phosphorylation levels. This is also how HER2 overexpression can magnify the signalling abilities of these other receptors, by enhancing the availability of HER2 receptors for hetero-dimerization [21]. Overexpression of these other family members has been noted as an escape mechanism during HER2 blocking, particularly HER3 which is so closely linked to PI3K/Akt signalling [22].

The oestrogen receptors, ERα and ERβ, are key drivers of a subset of breast cancers linked to poor prognosis and rapid proliferation of tumours [23]. There is evidence for the modulation of ER expression by HER2; interestingly, expression of HER2 and ER is frequently inversely correlated in breast cancer tumours. This modulation is likely enacted *via* the upregulation of Akt and MAPK signalling pathways which lead to the downregulation of ER transcription by the modulation of transcription factors [24, 25]. Progesterone receptors (PR) are steriod receptors that function as transcription factors when activated by progesterone. These receptors also impacts upon tumour behaviour in patients and can be coexpressed with HER2 and ER [26, 27].

### Breast cancer diagnosis and subtyping

Core biopsies are taken for all newly diagnosed and recurrent patients which are screened for the presence of HER2 using immunohistochemistry (IHC) and *in situ* hybridisation (ISH) techniques which correlate to likely benefit from targeted treatments [28–30]. IHC provides a score based on the intensity and completeness of membranous staining with a HER2-specific antibody that either confirms or rejects HER2 overexpression. For those patients with an intermediate score ISH testing is required [31]. Protein expression and gene amplification frequently, but not always corresponds [32]. For ISH a dual probe may be used for HER2 and CEP17 which is specific to the centromere region of chromosome 17. This allows for the correcting of non-polysomy amplification of chromosome 17 that may occur owing to chromosomal instability [33–35]. During this testing overexpression of oestrogen receptor and progesterone receptor are identified and the patient receives an ER, PR and HER2 status. ER and PR status is sometimes grouped and denoted hormone receptor (HR) status [36].

HER2 amplification can occur against different transcriptional backdrops. Sørlie et al. [37] identified intrinsic molecular subtypes based on gene expression that correspond with the molecular changes driving tumorigenesis and clinical features. Amplification of the *ErBb2* gene defines the HER2+ group with typically an ER-/PR- status. The luminal group exhibits an mRNA expression profile resembling non-cancerous luminal epithelial breast tissue. Luminal A typically is ER+/PR+/HER2- whereas luminal B tends to be HER2+, although complete segregation is not guaranteed [38]. Basal tumours have a gene signature alike to basal epithelial and normal breast myoepithelial cells. This group mostly comprises of ER-/PR-/HER2- patients but patients can express amplified *ErBb2* [39]. Normal-like tumours have patterns of expression like normal breast tissue and tend to be ER+/PR+/HER2- [40]. Gene expression deviations in these subtypes will impact upon HER2 signalling. For example, ER- and ER+ breast cancers show distinct transcriptional profiles and cross-talk occurs with ER [41] ER is able to activate HER2 signalling so HER2 activity may differ between HER2+ basal and luminal tumours [42].

**Figure 3:**
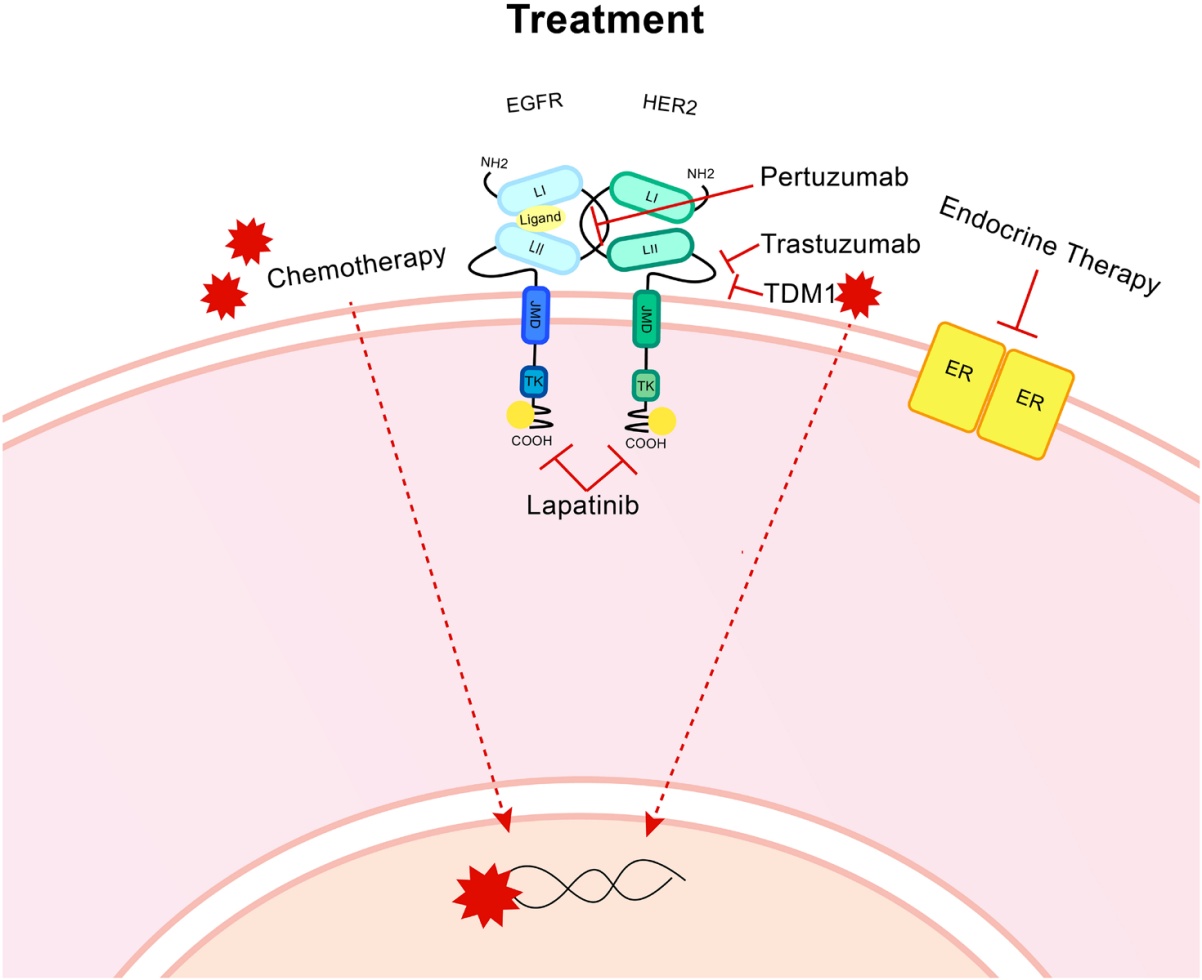
Treatment for HER2+ breast cancer. Targeted treatments include the humanised monoclonal antibody trastuzumab, which targets the CII extracellular domain of HER2. A second monoclonal antibody, Pertuzumab targets the binding domain CI that is also present on the extracellular region. Lapatinib is a dual inhibitor of both HER2 and EGFR and targets the intracellular region to block the cellular signal activating kinase activity. T-DM1 is an antibody drug conjugate which uses the trastuzumab monoclonal antibody to bind to the HER2 receptor then cell-receptor mediated endocytosis allows entry for the anti-microtubule DM1. Endocrine therapy is utilised for ER positive patients. Chemotherapy, radiotherapy and surgery are all utilised.

Chemotherapeutics, surgery, endocrine treatment (for ER+ tumours) and anti-HER2 therapies are administered based on biomarker status, metastatic profile, lymph node involvement and previous treatment response (Figure 3). HER2 targeted therapies can be highly successful, although resistance remains a problem, particularly when metastatic disease is evident.

### Her2 targeting treatments

Trastuzumab (Herceptin^®^) is a monoclonal humanised antibody that binds to the extracellular region of HER2 and exhibits multiple modes of anti-cancer activities [43]. Inhibition of downstream signalling through RAS/MAPK and PI3K/Akt produces a ligand-independent reduction in signalling, although whether this is due to a blocking of HER2 homodimerization is uncertain [18]. Cell cycle arrest at G_1_ occurs, likely in response to signalling changes. There is reduced shedding of the extracellular region of HER2 [44, 45]. Antibody-dependent cellular cytotoxicity is enhanced by the interaction of receptor-bound trastuzumab and Fc receptors present on immune cells, particularly T and B cells and natural killer cells [46, 47]. Binding of trastuzumab to HER2 causes a reduction of the receptor at the cell membrane *via* up-regulation of the normal endocytosis, which cycles receptors to and from the cell membrane at varying rates [48, 49]. Trastuzumab represented a great improvement in the treatment of HER2+ breast cancers and remains a common first-line treatment option [50, 51]. However, the development of resistance to trastuzumab is not uncommon and requires advancements in treatments and biomarkers [52].

Pertuzumab (Perjeta™) is a second humanised monoclonal antibody that binds to the extracellular region of HER2 at a site distinct to trastuzumab, so combination is non-competitive [45]. This binding region encompasses the dimerization site with EGFR and HER3, so ligand-dependent signalling activation is targeted. Dual use of pertuzumab and trastuzumab provides advanced progression free survival with chemotherapy [53]. Reduced phosphorylation of HER2, EGFR, HER3, ERK and Akt mediate a drop in proliferation and increase in apoptosis [44].

The small molecule dual inhibitor lapatinib (TYKERB^®^) targets both EGFR and HER2 by competing for the ATP-binding intracellular region blocking kinase activity and the activation of signalling pathways and causing cell cycle arrest [54]. Used in combination with trastuzumab, lapatinib can alter tumour phenotype and cause arrested cell cycling at G_1_ and a switch to a luminal phenotype. This switch may be problematic as HER2 targeted treatments will lose efficacy but by potentially increasing sensitivity to luminal A treatments like CDK4/6 inhibitors other treatment options may become viable [52]. Pertuzumab and lapatinib are often used in combination for those patients resistant to trastuzumab treatment [55, 56].

### Next generation treatments

Newer developments in anti-HER2 treatments attempt to widen the therapeutic index by reducing off-target effects to allow for increasing selectivity and high efficacy. T-DM1, (ado-trastuzumab emtansine or Kadcyla™) is an antibody-drug conjugate that combines trastuzumab and the cytotoxic DM1. The trastuzumab molecule binds to a HER2 receptor at the cell surface and the DM1 molecule can enter the cell *via* receptor-mediated endocytosis. Upon release from the lysosome mitosis is arrested, microtubules can no longer be assembled and apoptosis is triggered [57]. This drug was approved for use in HER2+ metastatic breast cancer in 2013 by the FDA [58].

Following from the success of T-DM1, T-DXd (fam-trastuzumab-deruxtecan-nxki [Enhertu]) was developed and approved for unresectable or metastatic HER2+ breast cancer patients who have previously received two or more HER2 targeting treatments [59]. This combines a HER2 antibody, a cleavable linker and the DNA topoisomerase I inhibitor, DXd [60]. Upon binding to HER2 T-DXd is internalised and the linker cleaved by lysosomal proteases to release DXd, which triggers cell death. Unlike DM1, DXd is cell membrane permeable and so adjacent cells are also targeted, which is an advantage in heterogeneous tumour environments where neighbouring HER2- cells may represent a resistance mechanism [61]. T-DXd does not suffer from linkage instability so the risk of off target effects is low and a higher antibody: drug ratio can be utilised. In phase two clinical trials the majority of patients, some who had received a great number of previous treatments, experienced stable antitumor activity [62].

Small molecule inhibitors continue to be utilised and developed, including neratinib [63]. This is a pan-HER irreversible tyrosine kinase inhibitor approved for extended adjuvant treatment in HER2+ breast cancer patients. It exhibits a small but significant improvement in survival for patients who have previously received trastuzumab and does not exhibit cross-resistance [64]. This drug can reduce signalling and induce apoptosis [65]. Lower incidences of central nervous system metastases may indicate this inhibitor should be used for patients at high risk of this metastatic pattern [66]. There is some evidence this inhibitor may be used to target mutations in the tyrosine kinase domain of the HER2 receptor, even in HER2- tumours [67].

Toxicity can be enhanced by EGFR targeting by dual HER2/EGFR treatments like neratinib. A highly selective tyrosine kinase inhibitor, tucatinib, has been developed. It does not inhibit EGFR, perhaps due to structural differences in the ATP-binding pocket [68]. This inhibitor can reduce activation of HER2, HER3 (through the downregulation of HER2/HER3 dimerization) and Akt resulting in reduced cell proliferation and tumour growth *in vivo* [69]. Treatment results in anti-tumour activity in patients who have previously received trastuzumab, pertuzumab, T-DM1 and lapatinib [70, 71]. Interest surrounds a positive response in patients with brain metastases [72]. Owing to the selective nature of this inhibitor combinations with other HER2-targetting agents may result in lower toxicity than other dual inhibitors [69]. Further advancements continue which may lead to the approval of further anti-HER2 treatments in breast cancer [68].

### Variants of HER2

At the present time, we have an incomplete understanding of why patients with HER2+ breast cancer exhibit variable responses or resistance to targeted therapies [73, 74]. One consideration is the structure of the HER2 protein, which is crucial for anti-HER therapy binding, and whether specific changes to its protein structure can influence patient responses to different targeted therapies [75]. We know that different forms of HER2 proteins are present in breast tumours and influence how breast cancer cells grow and spread throughout the body, as well as influencing patients responses to targeted therapies [76–80]. These forms can be produced *via* different changes to transcription and translation during protein production. During transcription, introns are typically spliced out of the transcriptome before the protein is produced during translation [76, 81]. Splice variants are produced when transcription is altered, frequently by the differential inclusion or exclusion of introns and exons [81, 82]. Changes to translation and post-translation modifications, such as alternative initiation of translation and proteolytic shedding can also produce different protein isoforms [83]. This review aims to summarise the current landscape of HER2 variant research and why we should consider HER2 variant levels and ratios when offering the best treatment plan for breast cancer patients.

## SPLICE VARIANTS OF HER2

### Δ16-HER2

**Figure 4:**
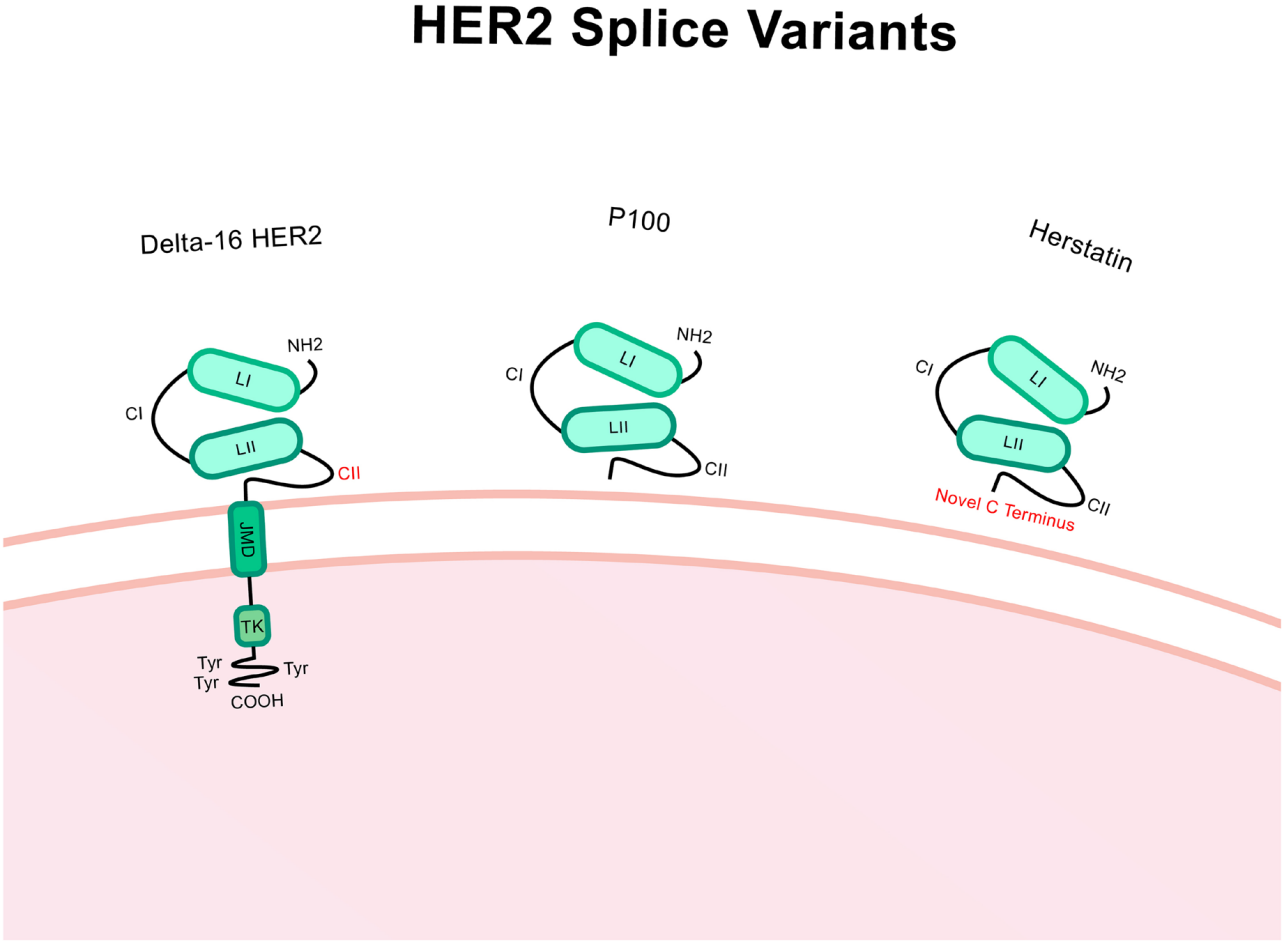
Structure of Δ16-HER2, P100 and herstatin splice variants. Compared to HER2 wild-type, Δ16-HER2 has a deletion of the exon 16 which encodes a 16 amino acid portion of the extracellular region within the CII area. P100 has the intron 15 retained which includes a premature stop codon which causes truncation and only the extracellular region of the protein is translated. Herstatin includes intron 8 which also has a premature stop codon and so again only the extracellular region is translated. The C terminus is altered in this case. Altered regions are highlighted in red.

Δ16-HER2 contains an in-frame deletion of the small cassette exon 16 producing a HER2 variant lacking 16 amino acids in the juxtamembrane region (Figure 4) [84]. Δ16-HER2 expression is common in breast cancer and typically represents approximately 9% of the complete HER2 wild-type (WT) transcript [85]. This exon deletion causes exposure of unpaired cysteine residues and the subsequent formation of cysteine bridges which triggers dimerization [86]. These dimers are constitutively activated and consistently remain present on the cell surface owing to the stable covalent bonding, enhancing downstream signalling [87]. So far, the splice factors SRSF3 and hnRNPH1 have been associated with splicing regulation of Δ16-HER2 [88].

Transgenic Δ16-HER2 mice suffer very rapid mammary carcinogenesis compared to HER2 WT mice. These tumours displayed altered vascularisation with HER2 WT-expressing tumours exhibiting a few large vessels and Δ16-HER2 mice numerous small vessels [89]. Δ16-HER2 mice also exhibit a distinct genetic profile and changes to the activation of various transcription factors [87]. A multifocal, metastatic profile with a heterogeneous expression of Δ16-HER2 is typical. Enhanced levels of pSrc in Δ16-HER2 expressing mice indicate a potential mechanism of action of heightened Src signalling that may drive this splice variants particularly oncogenic properties [90].

*In vitro* studies have associated high Δ16-HER2 expression with heightened Wnt and notch signalling, which are associated with tumour aggressiveness, breast cancer stem cell maintenance and EMT [91]. Increased motility, migration and the formation of invasive 3D structures were evident, even with relatively low levels of Δ16-HER2 [84]. Δ16-HER2 expression results in the activation of the Src signalling pathway *via* promoting the recruitment of Src kinase to the cell membrane followed by direct physical interaction [92].

A higher Δ16-HER2: HER2 WT ratio has preliminarily been linked to ER- tumours, high grade tumours and lymph node involvement at diagnosis [92]. Based on *in vitro* models, Δ16-HER2 was initially thought to enhance resistance to trastuzumab [85]. However, trastuzumab appears to be more effective at targeting Δ16-HER2 in murine models than HER2 WT [89]. Tilio *et al*. tested response to the anti-HER2 therapies; lapatinib, saracatinib (a Src inhibitor) and dacomitinib (an EGFR inhibitor) on Δ16-HER2 transgenic mice [93]. An intrinsic mode of resistance to lapatinib was identified *via* preferential binding to open conformation receptors, such as inactivated HER2 WT, suggesting the constitutively activated Δ16-HER2 is not effectively blocked. Acquired resistance to saracatinib treatment by the upregulation of ERK signalling has also been reported. Dacomitinib successfully blocked mammary tumour formation. Dasatinib (a Src inhibitor) has also led to a loss of function of Δ16-HER2 by disrupting interactions with Src kinase [92]. Alternatively, T-DM1 was not effective at treating cells transformed with Δ16-HER2, perhaps explained by the lack of internalisation of Δ16-HER2 dimers which is required for T-DM1 efficacy [87]. Analysis of primary tumours identified a link between heightened Src signalling, seen in high Δ16-HER2 expressing patients, and better response to trastuzumab [90]. Taken collectively it is therefore evident that Δ16-HER2 has important influence over tumour phenotype and treatment owing to its transforming properties and associated amplified cell signalling. Altered disease profile and response to treatments means the potential for measuring Δ16-HER2 expression to improve treatment choice could prove highly beneficial.

### Herstatin

Retention of intron 8 of ErbB2 produces the HER2 protein Herstatin, a secreted ‘auto-inhibitor’ [94]. Herstatin is a 68kDa secreted HER2 protein variant containing the extracellular subdomains, which are homologous to HER2 WT, followed by a novel 79 amino acid C-terminal region (Figure 4) [95]. It’s noteworthy that one study proposed that the presence of Herstatin transcript does not segregate by tumour grade or size, patient age, lymph node involvement or ER status and that mRNA transcripts were present in matched non-cancerous breast tissue and breast carcinomas [96].

Kolestsa *et al.* identified in non-cancerous samples, Herstatin was more prevalent in epithelial cells and mast cells, suggesting a paracrine mode of action [97]. In the breast carcinomas, the mRNA levels of Herstatin failed to predict for protein levels in 75% of the samples. This suggests a potential regulatory mechanism that blocks variant translation during tumorigenesis which serves to ‘protect’ the cancer cells from the auto-inhibitor. Within the remaining 25%, where high Herstatin protein levels were found, it may be that treatments to disrupt HER2 dimer formation may not be useful [96].

Herstatin binds to the HER2 WT cysteine-rich domain 1 (CI) using its novel C-terminus [98]. EGFR, HER4 and insulin-like growth factor 1 (IGF-1) can also bind with Herstatin [94]. Due to this binding ability Herstatin can block HER2 WT dimer formation, which earned it the title of ‘auto-inhibitor’. The ligand epidermal growth factor (EGF) could not induce EGFR dimerization when Herstatin was present [98]. Justman *et al.* produced a stably expressing Herstatin and EGFR cell line and identified a HER2 WT protein expression drop and that the receptor has reduced ability to produce homo- or hetero-dimers when Herstatin is expressed [99]. The phosphorylation of its tyrosine residues subsequently dropped. This produced a reduction in Akt signalling after stimulation with EGF and Transforming Growth Factor-α (TGF-α) which decreased proliferation. The MAPK pathway was unaffected in this instance [99].

*In vitro* studies identify a reduction in the trans-phosphorylation of HER3 by HER2 owing to Herstatin expression, exemplifying that this variant can impact upon a wider range of receptors than it can bind directly to. This influences cell survival, colony formation, anchorage independent growth and proliferation [96, 98]. Crucially, cells expressing high levels of Herstatin are more sensitive to Tamoxifen (a treatment for ER positive patients) [7].

### P100

Less is known about the truncated HER2 protein called P100. P100 lacks the intracellular domain of HER2 WT owing to retention of intron 15 which includes a premature termination codon and poly (A) addition site which triggers truncation (Figure 4). This variant can be secreted and it is hypothesized that P100 reduces efficacy of monoclonal antibody HER2 treatments by binding to them outside of the cell and reducing antibody binding to and blocking of HER2 WT [100]. *In vitro* studies have identified reduced proliferative abilities, even when treated with the ligand heregulins. Anchorage-independent growth was also reduced when P100 expression was induced in the HER2- ER+ cell line, MCF7. In this instance, downstream signalling *via* ERK1/2 was reduced as was HER4 phosphorylation [101].

## EXTRACELLULAR DOMAIN HER2 PROTEINS

### Carboxyl tail fragments and P95

Carboxyl tail fragments (CTFs) are a range of proteins 90–115 kDa in length that arise from proteolytic cleavage or alternative initiation of translation of the full length HER2 protein. Alternative initiation of translation produces 687-CTF and proteolytic cleavage produces 648-CTF and 611-CTF [102]. Protein cleavage and shedding occurs *via* metalloproteases, specifically the matrix metalloprotease activator 4-aminphenylmercuric acetate (APMA), which causes cleavage at the cell surface. A truncated protein capable of phosphorylation remains embedded in the membrane and the cleaved ECD is released [103].

The nomenclature for CTFs varies in the literature and they are typically collectively referred to as P95. One CTF, 611-CTF, has been a research focus. 611-CTF is a membrane bound and correctly folded protein meaning kinase function is retained. This is a particularly oncogenic CTF owing to the formation of stable dimers produced by disulphide bonding of exposed cysteine residues, much like Δ16-HER2 [104]. 611-CTF can active both Akt and ERK1/2 [105]. Independently, these key signaling nodes were recorded as increasing then decreasing with 611-CTF expression and pSrc as increasing to plateau. This pattern persisted even with low levels of expression indicating a relatively large impact per receptor molecule [104]. Also, advanced migratory ability and wound healing has been identified in 611-CTF expressing cell lines [106]. A lack of ER and PR expression correlates with 611-CTF positivity and down-regulating 611-CTF expression *in vivo* induced upregulation of ER [102].

611-CTF lacks the trastuzumab binding domain, indicating involvement in resistance. Molina *et al*. found *in vitro* trastuzumab blocked ECD shedding independently to the down-regulation of HER2 WT at the cell surface, trastuzumab was effective in this instance [103]. In contrast, a second study identified resistance to trastuzumab in a cell line stably transfected with 611-CTF compared to HER2 WT. Lapatinib treatment was effective, reducing phosphorylation and tumour volume and halting cells in the G_0_–G_1_ stage [105]. This difference may be explained by the combined natural expression of HER2 WT and CTFs in the first study which used HER2+ cell lines and singular stably transfected expression of either HER2 WT or 611-CTF in HER2- cells in the second.

Assessment of the predictive value of P95 for patient response to drug treatments has given conflicting results. In a cohort of primary HER2+ breast cancer patients treated with either chemotherapy and trastuzumab or lapatinib (or both combined), no correlation to 611-CTF expression was seen with neoadjuvant lapatinib or trastuzumab treatment. A correlation with increased ki-67 proliferation marker staining was seen [107]. Another early cancer cohort of HER2+ breast cancer patients treated with chemotherapy and trastuzumab found those with high 611-CTF had lowered overall survival and progression free survival. These patients were also twice as likely to suffer lung metastases [108].

In a cohort with metastatic cancer, 60% of HER2+ cases had high levels of 611-CTF (measuring the primary tumour site only) [109]. This expression correlated with high grade and HR negativity. In the group with HER2+/ER+ 611-CTF expression was predictive of lowered progression free survival and overall survival. In this cohort, 611-CTF expression did correlate with poor response to trastuzumab [109]. These differences may result from a lack of standardized assay and changes to cohort inclusion criteria. If multiple CTFs have been assessed as a P95 rather than a specific focus on 611-CTF a potential dilution of any effects on drug efficacy may have occurred. Multiple CTFs expressed in tandem may impact upon drug resistance in patients.

## CO-EXPRESSION OF HER2 VARIANTS

Tumours are heterogeneous, containing many cell types and a variable microenvironment. With the added potential for metastatic spread and secondary tumour sites, large differences in cellular level genetics will inevitably occur within and between different areas of a patient’s tumour (s) which may alter over time. Alternative splicing allows added plasticity for modification to important cellular functions, in the case of HER2 giving the opportunity to modulate signaling pathway activation that impacts upon cellular functions often associated with cancer-related phenotypes.

Previous studies have identified Δ16-HER2 and HER2 WT transcripts as being co-expressed at varying levels in breast carcinomas [85, 110]. The transcript of Herstatin appears to be downregulated in cancers potentially as a protective mechanism against its ‘auto-inhibitory’ abilities [96]. Multiple CTFs are also present in tumours, with 611-CTF focused upon as having oncogenic properties [102]. It is clear HER2 expression is not as simple as a single oncogenic overexpressed protein. It is likely many variants, arising from splicing and other mechanisms, are present in tandem. The relative ratios of these are likely to fluctuate depending on cellular conditions, during tumorigenesis and breast cancer progression. Variant expression may even drive certain breast cancer cases. Assessment of multiple HER2 variants at the transcript and protein expression level will improve our understanding of alterations to the HER2 status and ultimately the impacts this has on cell signaling and downstream effects. Studying splicing regulation and how this is altered in breast cancer could explain patterns of expression and how these link to treatment resistance [111].

## IMPLICATIONS OF STRUCTURAL ALTERATIONS FOR DIMERIZATION AND SIGNALING

The protein structures of EGFR, HER2, HER3 and HER4 are crucial in mediating dimerization and signaling and therefore alterations have major implications. Without the correct formation and folding of the protein, ligand binding and subsequent activation of the tyrosine kinase domain cannot occur [6]. Even small alterations to the extracellular domain can block homo- or hetero-dimerization and subsequently reduce the activity of not only HER2, but the other family members [112]. This will alter the activation of downstream signaling pathways, including those involved in cross-talk with other receptors [17, 18]. It is the structural differences of the C-terminus that gives specificity to the receptors, as the extracellular and kinase domains are conserved [113]. Ligands are specific to each receptor, with some cross-over, based on these structures [114]. The ligand binding causes a conformational shift that will favor a certain dimer formation with altered stability of the asymmetrical dimer formation depending on which ligand has bound [9, 115].

After HER2 is activated, adaptor proteins must be able to dock the newly phosphorylated tyrosine kinase domain to trigger the signaling pathways, including the RAS/MAPK and PI3K/Akt. Its structure also regulates any interaction with other cell surface receptors, like Mucin 1 [116]. These proteins include growth factor receptor-bound protein 2 which is the first step in the RAS/MAPK pathway and p85, which when docked releases p100, which can then activate the PI3K/Akt pathway [116].

The deletion caused by the alternative splicing event that produces Δ16-HER2 deletes a cysteine, leaving its partner exposed and able to produce disulphide bonds with a second receptor, including the other family members. These bonds are sufficient to produce strongly covalently-bound dimers that are not endocytosed with an active conformation and so constitutive signalling [117, 118]. It is these conformational alterations that mediate the oncogenic properties of this variant *via* the increased activating potential caused by this change in dimer bonding. In particular the Src, wnt and notch pathways are upregulated by expression of this splice variant as the kinase domain retains its open conformation [90]. P95 and the carboxyl tail fragments follow a similar pattern caused by exposed cysteine residues creating strong disulphide bonds increasing the activation of the RAS/MAPK and PI3K/Akt pathways [104].

For the truncated variant, Herstatin, disruption of dimerization by blocking the domains responsible for receptor interaction mediates the reduction in cell signalling [96]. A novel 79 amino acid C-terminal sequence binds to the CI domain of the HER2 WT receptor. As Herstatin does not have a kinase domain no phosphorylation of the HER2 WT receptor is achieved [94]. This inhibits docking of adaptor proteins, preventing all downstream signalling [98]. Currently, there is no direct evidence that P100 can bind to HER2 WT however, a reduction in ERK1/2 and HER4 phosphorylation may indicate a similar mechanism of signal blocking [101].

## VARIANT INFLUENCE ON PATTERNS OF DRUG RESISTANCE

Some causes of anti-HER2 treatment resistance are understood, such as the upregulation of other receptors to elevate signal blocking in HER2 addicted tumours [119, 120]. Signalling pathways may be activated *via* these receptors or other molecules, for example the loss of Phosphatase and tensin homolog (PTEN) enhances PI3K/Akt signalling despite HER2 blockage [56]. However, subsets of patients can have suboptimal responses to treatment without explanation.

HER2 variants have altered sensitivity to different HER2 targeted treatments owing to alterations in the protein structure at the sites of drug interactions. As mentioned above, Δ16-HER2 influences tumour aggressiveness and response to treatment. However, both trastuzumab and dasatinib are effective at treating this variant [89, 91]. Alternatively, as dimers containing Δ16-HER2 are not internalised, T-DM1 is not effective [87]. Those patients with high Δ16-HER2 may face a suboptimal response if given T-DM1 whereas use of trastuzumab could be highly effective. Alternatively, T-DXd contains the cell permeable drug DXd and cells expressing Δ16-HER2 adjacent to HER2 WT expressing cells may be targeted by the chemotherapy molecules, dubbed ‘the bystander effect’ [121]. As the tyrosine kinase domain is not altered inhibitors such as lapatinib, neratinib and tucatinib should retain efficacy and may provide prolonged reduction in signalling, as these receptors will remain at the cell surface [65, 69]. HER2 WT and Δ16-HER2 appear to be independently modulated during treatment [89]. If Δ16-HER2 remains high, owing to a potential driving mechanism in HER2+ tumours, this may present an opportunity for prolonging treatments that would normally decrease in efficacy during HER2 WT downregulation.

611-CTF is also a membrane bound variant of the HER2 receptor that can form the extremely stable dimers that confer the aggressive properties to Δ16-HER2. In some cases, this variant has been linked with a poor response to trastuzumab, likely owing to the truncation of the extracellular region where antibodies can bind [107–109]. Whether these correlations are reflective of a resistance conferred by the variant or simply a more aggressive cancer phenotype caused by more stable dimers is yet unknown. Again, the retention of the tyrosine kinase domain may allow successful treatment with inhibitors specific to this domain [103].

Secreted HER2 variants, including P100 and Herstatin, interact with HER2 targeting agents outside of the cell after secretion. As they maintain the CI and CII extracellular domains common to HER2 WT, binding to trastuzumab, pertuzumab, T-DM1 and T-DXd may occur, essentially expending the treatment prior to interaction at the cell surface [95, 122]. This is important to take into account as Herstatin can also restore sensitivity to ER targeting by Tamoxifen, indicating that tumours with high Herstatin levels may benefit from ER treatment despite HER2 WT signalling contributing to the development of ER signalling independence [7].

Work *in vitro* and *in vivo* as well as analysis from clinical trials has identified patterns of resistance to the standard of care treatment options in HER2+ patients which are correlated to variant expression. The focus currently remains on individual variants and HER2 WT. If patterns of variant expression could be identified and analysed against treatment response data in large patient cohorts a more detailed pattern of resistance may be recognised to support treatment recommendations.

## FREQUENT CONCURRENT MUTATIONS AND COMPLEMENTARY THERAPIES

Certain mutations and signalling pathway alterations frequently co-occur with HER2 overactivation, which can influence cancer phenotype and offer complimentary treatment options for HER2 positive patients [56]. The PI3K/Akt pathway is crucial for HER2 signalling and is driven predominantly through HER2/HER3 trans-phosphorylation [22]. PI3K inhibitors are available and may provide robust downregulation of signalling in combination with anti-HER2 treatments [123]. Mutations in the *PI3KCA* gene, which encodes the catalytic subunit of PI3K, have been linked to HER2 treatment resistance *via* upregulation of the PI3K/Akt pathway [17, 124, 125]. These are typically gain-of-function mutations that cause consistent activation of the PI3K/Akt pathway [126]. Where HER2 and *PI3KCA* mutations co-occur PI3K inhibitors have reinstated sensitivity to trastuzumab [127]. In combination with trastuzumab and TDM-1 inhibition of PI3K has been effective in advanced HER2+ breast cancer [128].

PTEN is a tumour suppressing dual phosphatase and an antagonist to the activation of Akt by PI3K. Trastuzumab treatment upregulates PTEN at the cell surface and increases activation *via* inhibition of Src activity [129]. PTEN loss is linked to a lack of response to trastuzumab and poor outcome [17, 56]. PTEN loss may be used to indicate a likely lack of response to treatment, particular trastuzumab [124, 125, 130].

Mammalian target of rapamycin (mTOR) can be activated by PTEN loss or *PI3KCA* mutations and is linked to trastuzumab resistance, again through the PI3K/Akt pathway. The mTOR inhibitor, everolimus, has been trialled in combination with trastuzumab and chemotherapy in HER2+ patients with advanced breast cancer which gave a moderate benefit to patients but increased toxicity [131]. The addition of the mTOR inhibitor, everolimus, can prolong progression free survival for patients with *PI3KCA* mutations, PTEN loss or hyperactivation of the PI3K/Akt signalling pathway but more specific inhibitors may need to be developed to reduce toxicity [130]. Biomarkers to determine the cause of PI3k/Akt pathway upregulation would enable clinicians to choose combinations of these inhibitors with anti-HER2 therapies to enhance prolonged response in patients [131].

*TP53* is one of the most frequently mutated genes in all cancers, including HER2+ breast cancer [126, 130, 132]. This tumour suppressor gene is a transcription factor that modulates genes involved in inhibition of cell cycling, apoptosis regulators, DNA repair pathway and inhibitors of angiogenesis and metastasis [133]. Mutations are most frequently found in the DNA-binding domain, resulting in a non-functional transcription factor [134]. Patients with HR- disease have greater incidence of *TP53* mutations [132, 135]. These mutations are linked to poor prognosis [136].

Cyclin D kinases (CDK) are a subgroup of serine/threonine kinases that can regulate cell cycle progression by interaction with cyclins to promote progression from the G_1_ to S phase [137]. Genetic and epigenetic alterations are frequently identified in cancers and high activity may supress cancer cell senescence [134]. Cyclin D1 mutations (of its gene *CCND1*) can cause hyperactivation of the MAPK pathway and so induce resistance to HER2 therapies [131]. *In vitro* inhibition of CDK4/6 has increased cell cycle arrest in synergy with trastuzumab and tamoxifen in HER2+ and ER+ cells respectively [137]. In ER+ cells, a highly selective CDK4/6 inhibitor (abemaciclib), was effective despite trastuzumab resistance [138]. A decrease in the phosphorylation of downstream effectors and evidence of single-agent and combination efficacy in HER2+/ER+ tumours has been identified. There was no change in response for patients with/without *PI3KCA* mutations but non-responders where more likely to have *TP53* mutations (in the p53 DNA binding domain) [134]. The monarcHER trial identified increased progression free survival for patients with advanced pre-treated HER2+ breast cancer with the addition of abemaciclib to trastuzumab in a chemotherapy-free setting [139]. Combining anti-HER2 therapies with those targeting downstream pathways has the potential to achieved prolonged suppression of HER2 pathways.

## HER2 VARIANTS AS BIOMARKERS

Variants of HER2 have been associated with key differences in tumorigenic potential, cancer behaviour and treatment response [79]. Assessing the ratios of these variants and establishing them as prognostic and predictive biomarkers would further personalise breast cancer treatment for HER2+ patients. Currently, upon diagnosis patients-derived biopsy tissue undergoes IHC staining to identify the expression status of ER, PR and HER2 as well as for the proliferation marker ki-67. HER2 positivity is measured on a score from 0 (HER2-) to 3+ (HER2+). For those intermediate tumours of a 2+ score, fluorescence *in situ* hybridisation (FISH) is employed to assess *ErbB2* gene amplification [32, 140]. Genomic testing can also be utilised to assess the expression of a panel of cancer-related genes and segregate tumours into subgroups based on intrinsic genetic expression patterns [141]. Groups are typically luminal A, luminal B, basal, HER2+ and normal-like. For example, the commercially available Mammaprint is used to assess HR+/HER2+ lymph node negative disease likelihood of benefiting from chemotherapy [142]. These systems are useful tools for doctors faced with deciding a patient’s treatment course, but they do have limits, both in the accuracy of the techniques and the limit of information gained from them [143]. Assessing both for HER2 status and HER2 variant expression could potentially refine prediction of patient response to treatment.

### Domain specific IHC

Currently, commercial IHC tests to assess HER2 protein levels target epitopes on the ECD, HercepTest™ being the most commonly used. HER2 status will only reflect the abundance of HER2 variants that include the particular epitope targeted. Patient samples that had previously been stained with a commercial IHC kit and diagnosed with a HER2 status were tested with anti-ICD and anti-ECD HER2 antibodies, which identified a subset of HER2- patients whose tumours exhibited phosphorylated HER2 ICD’s and activated Akt/MAPK pathways [144]. These patients may therefore have benefited from lapatinib treatment, which they did not receive following the initial diagnosis. A cohort of trastuzumab treated patients was assessed with an ICD and ECD antibody as well as FISH to assess gene amplification. Overall, gene amplification correlated more closely with the ICD antibody staining but labelling with the ECD antibody was more predictive of disease free survival after trastuzumab treatment [145]. Ratios of HER2 ICD to ECD are heterogeneous, both spatially in a tumour sample and because of treatment, which can cause reduction of HER2 at the cell surface or enhance shedding of the ECD. In paired core needle biopsy samples taken before and after treatment with trastuzumab and either docetaxel or pertuzumab, or both, ICD: ECD ratios changed in HER2+ and HER2- groups. However, this was not statistically significant [146]. Unexpectedly, trastuzumab binding was higher in tumours expressing high P95 levels than those with low P95 levels. As P95 lacks the trastuzumab-binding epitope, Recupero *et al*. suggests the loss of some HER2 ECDs on a cell surface increases access to full length HER2 proteins that could be blocked by essentially ‘freeing up’ space [147]. HER2 IHC testing is exceptionally useful when assessing a patient’s treatment course. Enhancing this diagnostic tool by testing ICD and ECD HER2 expression and the alteration of this throughout treatment would allow more accurate prescription of treatments. Further identification of specific variants that have been linked to increased drug resistance or sensitivity could follow from this. Of course, validation and thorough clinical trials of any new biomarker is required.

### mRNA assessment

mRNA assessment is already in use to segregate patients into subgroups that correspond to a particular gene expression pattern that has then been related back to patterns of response to available drug combinations [37]. This type of biomarker can be highly useful and recent advancements means the requirement for fresh frozen samples has been alleviated due to reduced cross-linking and degradation of mRNA [143]. In the case of splice variants, mRNA transcripts are distinct and could be assessed in patients. Further evaluation of HER2 splice variants as prognostic markers may produce a potential mRNA analysis capable of segregating tumours based on intrinsic genetic subtype that includes analysis of splice variants. This would aim to further personalise diagnosis with measurements of the ratios of splice variants to provide additional information on tumour behaviour and prospective treatment response.

## SERUM HER2 BIOMARKERS

Enzyme-Immunoassays can be used to assess levels of serum HER2 (sHER2) in the blood of patients. These assays measure the amount of secreted ECD HER2 produced by cleavage or splicing. It is noteworthy that patients suffering from comorbidities of hepatic diseases commonly have heightened sHER2 and are not applicable for this biomarker. Cohort studies have identified sHER2 testing as a useful complementary test to IHC owing to the correlations between high sHER2 and aggressive tumour phenotypes such as invasion and metastases. Heightened levels were also reported to be predictive of relapse and metastases to the bone, liver, and brain. A complete pathological response (pCR) was more likely in patients who exhibited a drop in sHER2 values after treatment with neoadjuvant trastuzumab [148]. Likewise, another cohort of HER2+ patients was more likely to achieve pCR when initial sHER2 levels were high and a drop in sHER2 was identified after neoadjuvant lapatinib treatment with chemotherapy. However, a portion of patients had increased sHER2 after lapatinib treatment [149]. When effective, trastuzumab can reduce cleavage of HER2 by blocking the cleavage sites required by metalloproteases. In those patients that responded well to treatment this may have explained the decrease in sHER2 [103]. Lapatinib may actually increase the sHER2 when effective as it has been identified as increasing ECD shedding [108].

Serum HER2 testing will not replace IHC for diagnostic testing in breast cancer patients but may provide added prognostic information and be predictive of treatment response in the cases of neoadjuvant trastuzumab and lapatinib. Monitoring may be a non-invasive indicator for relapse and the requirement of thorough patient screening. Further studies to identify correlation with response to a wider range of treatment options and a greater understanding of the biological implications of fluctuating sHER2 levels will enhance the potential of this biomarker.

### Clinical implications of biomarker development

Current practice entails testing for the overexpression of HER2, which if positive, suggests the use of trastuzumab plus pertuzumab and a taxane with the addition of T-DM1 in a second line setting [62]. Development of biomarkers to include HER2 variants could enable oncologists to prescribe personalised treatment based on the information available surrounding likely sensitivity to different treatments, and structural alterations that would block binding to the receptors. Crucially, any changes to these amounts may indicate the development of resistance and so recommend a change in treatment before the patient’s disease begins to progress. This could help with the choice of third line treatments of which there is no officially recommendation, although the use of combinations with lapatinib is common [150]. If IHC biomarkers, including domain specific antibodies, allow for the detection of HER2 variants with alterations to the binding domains of antibodies and tyrosine kinase inhibitors, these could be chosen prior to resistance developing [145]. If a patient had high levels of intracellular truncated variants, such a 611-CTF, lapatinib, neratinib or tucatinib could be chosen to compliment treatment with trastuzumab in a first line setting to pre-empt resistance through continued signalling [105].

mRNA expression of variants could also support clinical decision making by identifying ratios of expression of splice variants against, a backdrop of transcriptional subtype, to recommend various treatment choices [102, 110]. With the tracking of serum HER2 levels any increase in HER2 shedding may indicate resistance is developing and a tyrosine kinase inhibitor could be introduced to block signalling by intracellular HER2 receptors. As trastuzumab reduces shedding a combination can be used [148]. Clinical trials that integrate biomarkers into their designs are required [150]. Pair these developments with the tracking of other commonly mutated genes and a range of combination therapies may be chosen from to achieve prolong patient response to treatment [135].

## SUMMARY

The production of anti-HER2 therapies have greatly improved survival outcomes for breast cancer patients. A more complete understanding of the protein products of the *ErbB2* oncogene provides opportunities to personalise treatment. It is now understood HER2 variants can alter response to anti-HER2 treatments which may provide the opportunity to switch to more effective therapies. Future work should be focused at examining multiple variants of HER2 in tandem. Expanding breast cancer biomarkers to include changes to the HER2 protein and mRNA transcripts could improve patient diagnosis and provide more specific treatment recommendations.
